# Chemotherapy dose per kilogram lean body mass increased dose-limiting toxicity event in male head and neck cancer with taxane and platinum-based induction therapy

**DOI:** 10.1186/s12885-022-10152-y

**Published:** 2022-10-21

**Authors:** Chuan-Jen Hung, Bor-Hwang Kang, Keng-Ming Chang, Ying-Ying Kang, Chun-Hao Yin, Ching-Chih Lee

**Affiliations:** 1 Department of Otolaryngology, Head and Neck Surgery, Kaohsiung Veterans General Hospital, No.386, Dazhong 1st Rd., Zuoying Dist., Kaohsiung City, 81362 Taiwan (R.O.C.); 2 Department of Pharmacy, Tajen University, Pingtung, Taiwan; 3 School of Medicine, National Defense Medical Center, Taipei, Taiwan; 4 Department of Otolaryngology, Head and Neck Surgery, Tri-Service General Hospital, Taipei, Taiwan; 5 Department of Pharmacy, Kaohsiung Veterans General Hospital, Kaohsiung, Taiwan; 6 Institute of Clinical Pharmacy and Pharmaceutical Sciences, College of Medicine, National Cheng Kung University, Tainan, Taiwan; 7 Department of Medical Education and Research, Kaohsiung Veterans General Hospital, Kaohsiung, Taiwan; 8 Institute of Hospital and Health Care Administration, National Yang Ming Chiao Tung University, Taipei, Taiwan

**Keywords:** Head and neck cancer, Induction chemotherapy, Body composition factor, Chemotherapy dose per kilogram lean body mass

## Abstract

**Background:**

This study aimed to determine whether drug doses per kilogram of lean body mass (LBM) were associated with dose-limiting toxicity (DLT) events in head and neck cancer (HNC) patients.

**Methods:**

This retrospective cohort study included 179 HNC patients who underwent induction chemotherapy (IC) at a medical center from May 1, 2014, to May 31, 2021. HNC patients’ characteristics, tumor factors, IC regimen and dose, laboratory data, and body composition factors, including lean body mass (LBM) and skeletal muscle index (SMI), derived from CT, MRI, or PET scan images and drug dose per kilogram LBM were recorded. Dose-limiting toxicity (DLT) events were regarded as the primary outcome. Multivariate logistic regression was used to establish a novel risk score for DLT events by the abovementioned variables. The above-mentioned risk score was validated in another cohort.

**Results:**

The overall DLT events during the first cycle of IC for 179 HNC patients was 24%. After stratifying by gender, docetaxel per kilogram LBM > 2.52 mg/kg (adjusted odds ratio [aOR]: 3.18; 95% confidence interval [CI], 1.25–8.09), pre-treatment glutamic pyruvic transaminase (GPT) > 40 U/L (aOR, 2.61; 95% CI, 1.03–6.64), and history of chronic liver diseases (aOR, 3.98; 95% CI, 1.03–15.46) were significant variables in male HNC patients. The DLT events risk was categorized by summation of the above-mentioned risk factors for male HNC patients. Three risk groups were stratified by overall event of 17.6%, 25.8%, and 75%. The above-mentioned risk score had an acceptable discriminatory ability in another validation cohort.

**Conclusions:**

Among male HNC patients treated with IC, docetaxel per kilogram LBM more than 2.52 mg/kg, pre-treatment GPT > 40 U/L, and history of chronic liver disease were significant risk factors for DLT events. Identifying high-risk patients could help physicians prevent severe/fatal complications among HNC patients undergoing IC, especially for the male individuals.

**Supplementary Information:**

The online version contains supplementary material available at 10.1186/s12885-022-10152-y.

## Background

Head and neck cancer (HNC) is one of the most common and lethal cancers worldwide [1, 2]. Despite considerable improvements in treatment, such as radical resection, radiotherapy techniques, targeted therapy, and immunotherapy, HNC remains notorious for its high recurrence rates and distant metastasis rates [3, 4]. Recently, induction chemotherapy (IC) has been increasingly utilized. IC could be followed by definitive radiotherapy with or without chemotherapy or surgery during an organ preservation strategy in oropharyngeal, laryngeal, or hypopharyngeal cancer. After the improvements in primary care, an increasing number of promising studies have revealed IC’s protective effects, including decreased distant metastasis rates, improved survival rates, and organ preservation [5, 6].

Treatment-related toxicities, such as severe mucositis, febrile neutropenia (FN), acute kidney injury (AKI), and hyponatremia, are also increasingly being reported [7, 8]. Dose-limiting toxicity (DLT) event could lead to therapy dose reduction or delay and resulted in worse outcomes in turn [9]. Besides traditional factors, like comorbidities, performance status, and impaired renal or hepatic function, body composition factors, and related variables, such as chemotherapy per kilogram lean body mass (LBM) were reported to be associated with DLT [10, 11]. Low skeletal mass or sarcopenia was associated with increased chemotherapy dose-limiting toxicity (DLT) in HNC with chemoradiotherapy [12]. Chemotherapy dose per kilogram LBM had been reported to be significant predictors for toxicity event or DLT in lung cancer and colon cancer [13, 14]. However, its validity in HNC with induction chemotherapy was not explored.

In order to achieve adequate relative dose intensity (> = 80% at least) in chemotherapy, the first step was to identify high-risk populations for DLT and launch prevention protocols, like prophylactic G-CSF injection, more intensive care, or close follow-up [9, 15]. We aimed to perform a comprehensive analysis, consisting of chemotherapy dose per kilogram LBM, body composition factors, patients’ characteristics and tumor factors, laboratory data, etc., and develop a prediction model for DLT events in HNC patients with IC using a cancer registry database and clinical research database at our institution.

## Methods

### Patient population and treatment

**Fig. 1:**
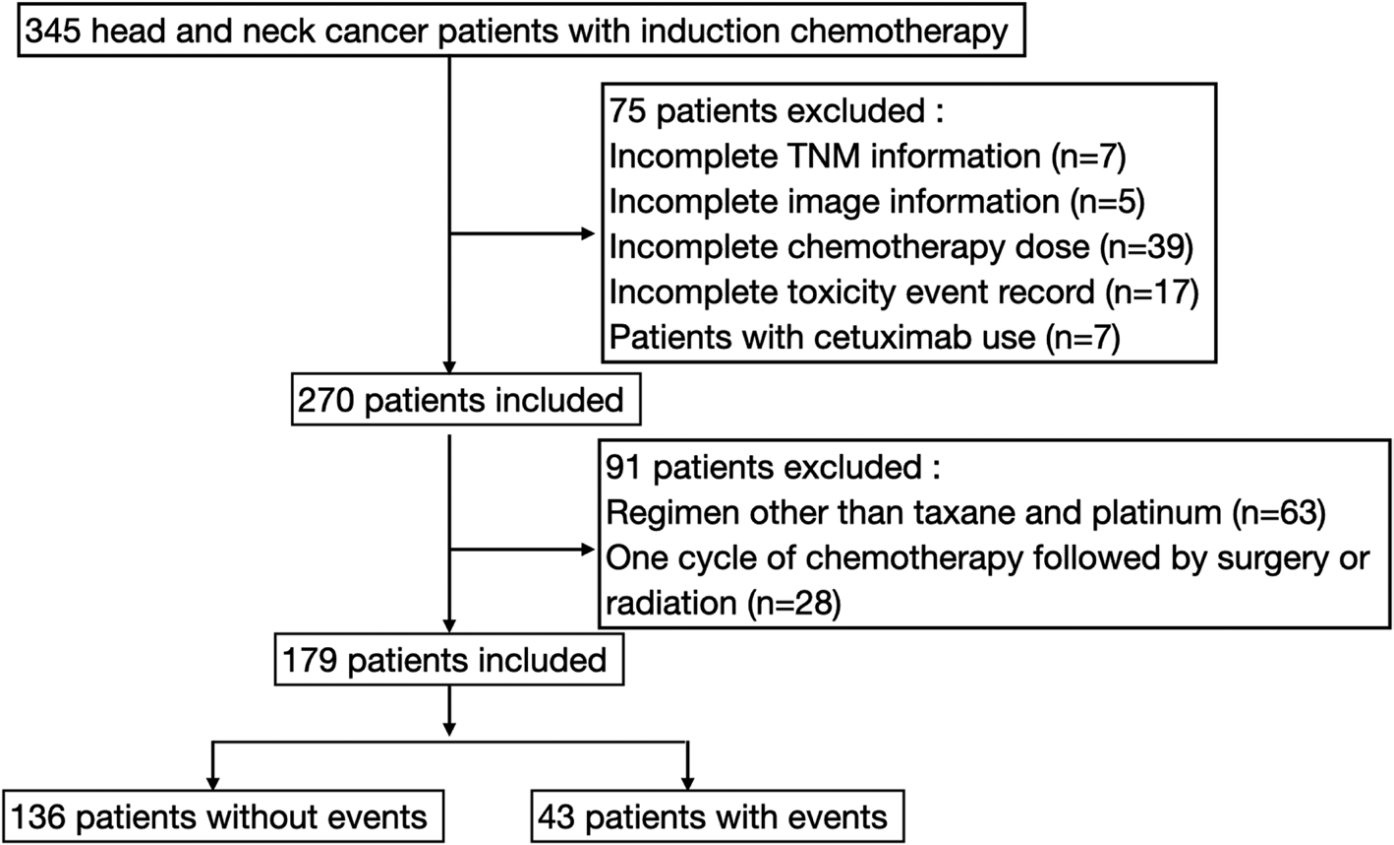
Study flow diagram

From the Cancer Registry Database of Kaohsiung Veterans General Hospital, 179 HNC patients who underwent taxane and platinum-based IC between May 1, 2014, to May 31, 2021, were identified (Fig. 1). IC was administered if a patient was under suitable conditions and had an Eastern Cooperative Oncology Group (ECOG) performance status score of 0–2. Our hospital has two main chemotherapy regimens, abbreviated as TP, and TPF. All regimens were administered every three weeks for two to three cycles. Patients who were scheduled for two to three cycles failed to undergo subsequent chemotherapy due to adverse events were also included for analysis. The TP regimen was the most used and consisted of docetaxel (60 mg/m^2^ on Day 1) and cisplatin (60 mg/m^2^ on Day 1). The TPF regimen was TP plus 5-fluorouracil (500 mg/m^2^ as a 4-day continuous infusion). For patients with poor renal function, cisplatin was replaced by carboplatin. After IC, no prophylactic antibiotics were prescribed unless severe toxicity occurred. The patient was re-evaluated using imaging studies to identify their overall response and determine the treatment course after two to three IC cycles.

The independent variables were categorized into three domains. One domain included the patients’ characteristics (age, sex, comorbidities, etc.) and tumor features (sub-sites, clinical TNM stages recommended by the American Joint Committee on Cancer [7^th^ edition before 2018, and 8^th^ edition thereafter] [16, 17], etc.). The second domain also included clinical laboratory data (complete blood cells and differential count, sodium, potassium, glutamic oxaloacetic transaminase (GOT), glutamic pyruvic transaminase (GPT), total bilirubin, albumin, glucose level, blood urea nitrogen, creatinine, etc.). Another domain included body composition measurements, including lean body mass (LBM) and skeletal muscle index (SMI). A third domain had chemotherapy dose and dose per LBM. SMI was derived from a cross-sectional area (CSA) at the third cervical vertebra of the pre-treatment computerized tomography (CT), magnetic resonance imaging (MRI), or positron emission tomography (PET) scan images, as previously described [18, 19]. Informed consent was not required due to the use of non-identifiable records. The Ethics Committee of our Institutional Review Board approved the study protocol (KSVGH22-CT1-03).

### Body composition measurements and chemotherapy dose per kilogram lean body mass

Body composition was estimated using the method developed by Swartz et al. [20]. The skeletal muscles CSA was measured on the reference slice as the first axial view where all vertebral arcs could be identified, along with the transverse and spinous processes. The measurement was taken by scrolling from the caudal to the cephalad direction on the head-and-neck images. The skeletal muscles CSA was manually determined twice by a single physician (Supplementary Fig. 1). The skeletal muscles CSA at the C3 vertebra (in square centimeters [cm^2^]) was determined as the sum of separate measurements of the paravertebral and bilateral sternocleidomastoid muscles. The intra-rater reliability correlation coefficient for CSA at C3 was acceptable (0.94; 95% confidence interval [CI], 0.923–0.953) (Supplementary Table 1). The following formulae were also used to calculate CSA, SMI, and LBM:

$$\mathrm{CSA}\;\mathrm{at}\;\mathrm L3\;(\mathrm{cm}^2)=27.304+1.363\times\mathrm{CSA}\;\mathrm{at}\;\mathrm C3\;(\mathrm{cm}^2)-0.671\times\mathrm{age}\;(\mathrm{years})+0.64\times\mathrm{weight}\;(\mathrm{kg})+26.442\times\mathrm{sex}\;(\mathrm{wherein}\;\mathrm{sex}=\;1\;\mathrm{for}\;\mathrm{females}\;\mathrm{and}\;2\;\mathrm{for}\;\mathrm{males})$$

$$\mathrm{SMI}\;\mathrm{at}\;\mathrm L3\;(\mathrm{cm}^2/\mathrm m^2)\;=\;\mathrm{CSA}\;\mathrm{at}\;\mathrm L3\;(\mathrm{cm}^2)/\mathrm{squared}\;\mathrm{height}\;(\mathrm m^2)$$

$$\mathrm{LBM}\;(\mathrm{kg})=0.3\times\mathrm{CSA}\;\mathrm{at}\;\mathrm L3\;(\mathrm{cm}^2)+6.06$$

Chemotherapy dose per kilogram lean body mass was determined as:

$$\mathrm{Docetaxel}\;\mathrm{dose}\;\mathrm{per}\;\mathrm{kilogram}\;\mathrm{lean}\;\mathrm{body}\;\mathrm{mass}\:=\:\mathrm{Docetaxel}\;\mathrm{dose}\;(\mathrm{mg})/\mathrm{LBM}(\mathrm{kg})$$

$$\mathrm{Cisplatin}\;\mathrm{dose}\;\mathrm{per}\;\mathrm{kilogram}\;\mathrm{lean}\;\mathrm{body}\;\mathrm{mass}\:=\:\mathrm{Cisplatin}\;\mathrm{dose}(\mathrm{mg})/\mathrm{LBM}(\mathrm{kg})$$

### Dose-limiting toxicity events and patient follow-up

The dose-limiting toxicity (DLT) events included (1) grade 3 or higher acute hematological adverse events like neutropenia (absolute neutrophil count [ANC] < 1000 with or without fever), anemia (hemoglobin < 8 mg/dL, or with life-threatening consequences), and thrombocytopenia (platelet < 25,000/μL), and non-hematological adverse events, such as acute liver injury (total bilirubin > 1.5_×_upper limit of normal (ULN); GOT or GPT > 5 _×_ ULN) and acute kidney injury (creatinine > 3_×_ ULN) according to the Common Terminology Criteria for Adverse Events version 5.0 or death after IC (https://ctep.cancer.gov/protocoldevelopment/electronic_applications/docs/CTCAE_v5_Quick_Reference_5x7.pdf), (2) death after chemotherapy, (3) subsequent dose reduction of >  = 50% for taxane or platinum, or (4) postponement of subsequent therapy of ≥ 4 days due to bone marrow suppression [14]. DLT events after the first cycle of induction chemotherapy was defined as the study endpoint.

Responses after 2–3 cycles of induction chemotherapy were analyzed according to Response Evaluation Criteria in Solid Tumors (RECIST) 1.1 criteria by CT/MRI images. Subsequent treatment modality was also reported.

### Statistical analyses

SAS version 9.4 (SAS, Inc., Cary, NC) was used to analyze the data. Categorical variables, such as sex, primary tumor site, clinical stage, and comorbidities, were analyzed using Pearson’s chi-square or Fisher’s exact test. Continuous variables, such as age, LBM, SMI, and chemotherapy dose per LBM, were compared using one-way ANOVA. Univariate logistic regression was performed at first, and variable with a *P*-value less than 0.1 were candidates for multivariate analysis. Multivariate logistic regression with backward stepwise method was applied to determine the significant variables. Rounded number of beta coefficient or summation of risk factors in the final model was used to establish a prediction model. Another cohort composed of 34 HNC treated with taxane and platinum-based induction chemotherapy between June 1, 2021, and May 31, 2022, were recruited in order to validate the above-mentioned risk score. A two-sided *P*-value < 0.05 was considered statistically significant.

## Results

### Dose-limiting toxicity events in HNC treated with induction chemotherapy

A total of 179 HNC patients who underwent IC with taxane and platinum were recruited. The mean age was 56.5 ± 8.8 years, and 91.1% were male. The study population consisted of 37 (20.7%) patients with oral cancer, 83 (46.4%) with oropharyngeal cancer, 51 (28.5%) with laryngeal/hypopharyngeal cancer, and 8 (4.5%) with other cancers, such as sinonasal cancer (Table 1). The mean estimated LBM and SMI was 40.2 ± 6.6 kg and 47.9 ± 7.3 cm^2^/m^2^, respectively. A large variation of docetaxel per kilogram LBM and cisplatin per kilogram LBM from 1.53 mg/kg to 4.14 mg/kg was observed compared to other body composition factors (Supplementary table 2). DLT events during the first cycle of IC was summarized in Table 2. 43 DLT events (24%) were recorded; 35 patients (19.6%) with grade III-IV adverse events, eight patients with death (4.5%), and nine patients (5%) with the postponement of subsequent chemotherapy.

**Table 1:** Demographic data (*n* = 179)

Variables	Number (%)
Age (mean ± SD)	56.5 ± 8.8
Gender	
Male	163 (91.1%)
Female	16 (8.9%)
Lean body mass (mean ± SD)	40.2 ± 6.6
Skeletal muscle index(mean ± SD)	47.9 ± 7.3
Docetaxel per kilogram lean body mass (mean ± SD)	2.53 ± 0.39
Cisplatin per kilogram lean body mass (mean ± SD)	2.53 ± 0.41
Clinical T classification	
cT1-2	58(32.4%)
cT3-4	121(67.6%)
Clinical N classification	
cN0-1	61 (34.1%)
cN2-3	118 (65.9%)
Tumor subsites	
Oral cavity	37 (20.7%)
Oropharynx	83 (46.4%)
Hypopharynx/Larynx	51 (28.5%)
Others	8 (4.5%)
Comorbidities	
Chronic liver disease	11 (6.1%)
Hypertension	27(15.1%)
Diabetes	17 (9.5%)
Induction chemotherapy regimen	
Docetaxel + Cisplatin	169 (94.4%)
Docetaxel + Cisplatin + 5-Fluorouracil	10 (5.6%)

**Table 2:** Frequency of toxicity events after the first cycle of induction chemotherapy (*n* = 179)

Variables	Number	%
Grade III + hematological adverse events	35	19.5
Neutropenia	34	19
Anemia	2	1.1
Thrombocytopenia	0	0
Grade III + non-hematological adverse events	1	0.6
Acute liver injury	0	0
Acute kidney injury	1	0.6
Grade III + hematological and non-hematological adverse events	35	19.6
Subsequent chemotherapy dose reduction more than 50%	0	0
Postponement of chemotherapy > = 4 days	9	5
Death during or after chemotherapy	8	4.5
Overall DLT events	43	24

*Abbreviation: DLT events* Dose-limiting toxicity events

### The association between chemotherapy dose per kilogram LBM and DLT in male and female patients

The distribution of body composition factors, chemotherapy dose per kilogram LBM, and DLT events between male and female patients were summarized in Table 3. Male patients had higher LBM (41.4 kg vs 28.5 kg; *P* < 0.001), and higher SMI (48.9 cm^2^/m^2^ vs 37.4 cm^2^/m^2^; *P* < 0.001). There was no statistical difference between the DLT event rate between male and female patients.

**Table 3:** Distribution of body composition factors and dose-limiting toxicity events in male and female head and neck cancer patients

Variables	Males (*n* = 163)	Females (*n* = 16)	*P* value
*Body composition factors and chemotherapy dose per kilogram LBM*			
BMI	23.6 ± 4.1	22.5 ± 3.1	0.901
LBM	41.4 ± 5.5	28.5 ± 4.5	< 0.001
SMI	48.9 ± 6.6	37.4 ± 5.4	< 0.001
Docetaxel/LBM	2.5 ± 0.31	3.3 ± 0.33	< 0.001
Cisplatin/LBM	2.45 ± 0.31	3.3 ± 0.33	< 0.001
*Outcome*			
DLT events	41(25.2%)	2(12.5%)	0.258

*Abbreviation: BMI* Body mass index, *LBM* Lean body mass, *SMI* Skeletal muscle index, *DLT events* Dose-limiting toxicity events

### Risk score for DLT events in HNC patients

**Fig. 2:**
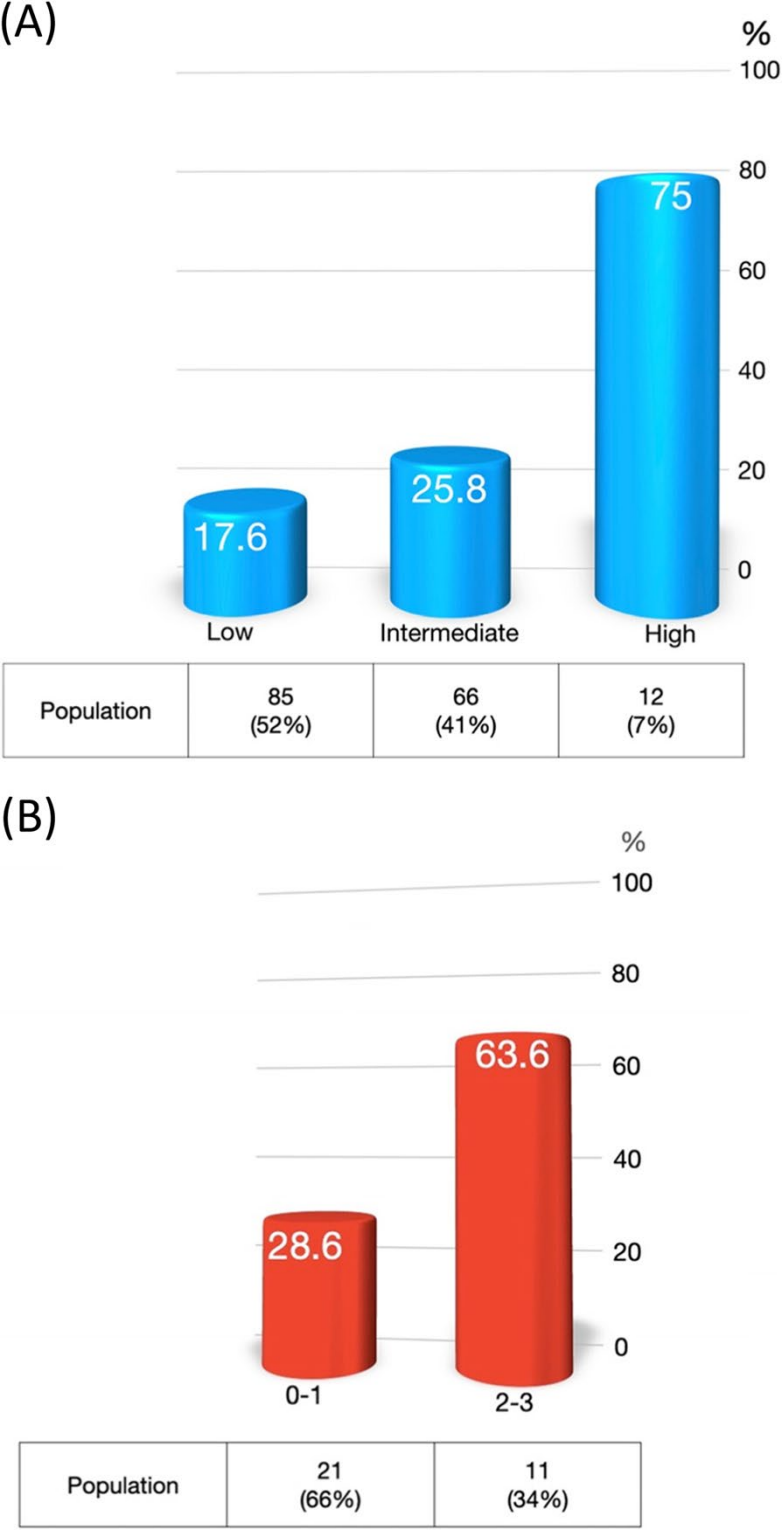
Dose-limiting toxicity event rate among low (0 factor), intermediate (1 factor), and high-risk (> = 2 factors) groups based on three risk factors in the derivation group (**A**), and validation group (**B**)

Due to the significant difference of body composition factors and chemotherapy dose per kilogram LBM between male and female patients, we performed univariate and multivariate analysis stratified by gender (Table 4). Due to a large difference of chemotherapy dose per kilogram LBM between male and female patients, different cutoff points were used (Supplementary table 3). Among male HNC patients, docetaxel per kilogram LBM more than 2.52 mg/kg (aOR = 3.18; 95% CI, 1.25–8.09), pre-treatment GPT more than 40 (aOR = 2.61; 95% CI, 1.03–6.64), and history of chronic liver disease (aOR = 3.98; 95% CI, 1.03–15.46) were significant predictors for DLT events in multivariate analysis. The beta coefficient for the abovementioned factors was similar (Supplementary table 4), and these factors could be added together for further risk estimation. There was no significant predictor for female HNC patients. Figure 2A illustrated the association of DLT events by summation of risk factors in male HNC patients. The DLT rate was 17.6% for the low-risk group (with 0 risk factor), 25.8% for the intermediate-risk group (with one risk factor), and 75% for the high-risk group (with two to three factors).

**Table 4:** Univariate and multivariate analysis of DLT events in male and female head and neck cancer patients

Variables	Male (*n* = 163)		Female (*n* = 16)	
	OR (95% CI)	aOR (95% CI)	OR (95% CI)	aOR (95% CI)
Lean body mass (kg)	0.94 (0.88–1.01)		1.15 (0.83–1.59)	
Skeletal muscle index (cm^2^/m^2^)	0.95 (0.9–1.01)		1.08 (0.81–1.42)	
Docetaxel per kilogram lean body mass (mg/kg)^a^				
Lower third	1	1	1	
Middle third	1.43 (0.55–3.74)	1.52 (0.57–4.01)	-	
Upper third	2.86 (1.16–7.04)	3.18 (1.25–8.09)	1 (0.05–22)	
Cisplatin per kilogram lean body mass (mg/kg)^a^				
Lower third	1		1	
Middle third	1.43 (0.55–3.74)		-	
Upper third	2.86 (1.16–7.04)		1 (0.05–22)	
Age	0.99 (0.95–1.03)		0.86 (0.72–1.04)	
Tumor stage				
cT3-4 vs cT1-2	0.69 (0.33–1.43)		0.4 (0.02–8.07)	
cN2-3 vs cN0-1	2.01 (0.88–4.59)		1 (0.05–19.36)	
Tumor subsites				
Oral cavity	1		-	
Oropharynx	1.46 (0.62–3.43)		-	
Hypopharynx/Larynx	2.05 (0.84–5.04)		-	
Others	0.79 (0.09–7.25)		-	
Comorbidities				
Chronic liver disease	3.25 (0.89–11.86)	3.98 (1.03–15.46)	-	
Hypertension	0.71 (0.25–2.03)		-	
Diabetes	0.40 (0.09–1.82)		-	
Laboratory data				
WBC (× 1000/uL)	0.98 (0.89–1.09)		0.20 (0.02–2.06)	
Hemoglobin (g/dL)	1.03 (0.84–1.25)		1.77 (0.47–6.6)	
Platelet (× 1000/uL)	1.00 (0.99–1.00)		0.99 (0.98–1.02)	
BUN (mg/dL)	0.97 (0.91–1.04)		052 (0.14–1.96)	
Creatinine (mg/dL)	0.52 (0.051–5.2)		-	
eGFR (ml/min/1.73m^2^)	1.01 (0.99–1.03)		0.99 (0.9–1)	
Sodium (mEq/L)	1.03 (0.92–1.15)		1.32 (0.67–2.60)	
Potassium (mEq/L)	0.95 (0.5–1.8)		-	
Albumin (g/dL)	0.75 (0.32–1.79)		-	
Abnormal GOT^b^	2.59 (0.90–7.45)		-	
Abnormal GPT^b^	2.30 (0.94–5.63)	2.61 (1.03–6.64)	-	
Abnormal total bilirubin^b^	-		-	
Hematoloigcal markers				
Neutrophil to lymphocyte ratio	1.06 (0.9–1.14)		0.74 (0.28–1.96)	
CRP/Albumin	1.05 (0.57–1.93)		1.32 (-)	
mGPS				
mGPS 0	1			
mGPS 1	0.54 (0.18–1.6)		-	
mGPS 2	1.69 (0.49–5.77)		-	

*Abbreviation:* OR Odds ratio, CI Confidence interval, aOR Adjusted odds ratio, LBM Lean body mass, BUN Blood urea nitrogen, eGFR estimated glomerular filtration rate, GOT Glutamic oxaloacetic transaminase (GOT), GPT Glutamic pyruvic transaminase, CRP C-reactive protein, mGPS modified Glasgow Prognostic score

^a^ The cutoff points were different between the male and female patients

^b^ Abnormal GOT was defined as GOT > 40 U/L; Abnormal GPT was defined as GPT > 40 U/L; Abnormal total bilirubin was defined as total bilirubin > 1.2 mg/dL

- Estimated data can’t be convergent

### Validation of risk score

Another cohort composed of 34 HNC patients was used to validate the risk score (supplementary table 5). The demographic data between the derivation group and validation group was similar. DLT events developed in 13 (38.2%) patients (supplementary table 6). Due to limited cases in the validation group, the patients were categorized into two groups. Among male HNC patients, the DLT event rates for score 0–1 and 2–3 groups were 28.6% vs. 63.6%, respectively (*P* = 0.055) (Fig. 2B).

## Discussion

This study identified novel predictors of IC toxicities in HNC using a verified cancer registry database. During the first cycle of taxane and platinum-based IC in HNC, up to 24% DLT events were recorded in our series. Docetaxel per kilogram LBM, pre-treatment GPT > 40 U/L, and history of chronic liver disease were strongly associated with DLT events in male patients. The above-mentioned prediction model was validated with another cohort. For male HNC treated with IC, the abovementioned variables should be identified, and high-risk group deserved closer follow-up and care.

Compared to other studies, our research has several strengths. The prediction ability of chemotherapy dose per kilogram LBM was first validated in male HNC patients with taxane and platinum-based induction chemotherapy for DLT events [13]. Although previous literature had used a similar concept, its application for toxicity event prediction was not widespread [14]. Moreover, we have performed a comprehensive analysis for DLT events. Variables like patients’ characteristics, laboratory data, inflammation markers, body composition factors, and chemotherapy dose were all included. In addition, the correctness of our data was validated. The Cancer Registry System in Taiwan is supervised by the Health Promotion Administration of the Ministry of Health and Welfare. They regularly monitor data correctness and implement the accreditation of cancer centers every three years (https://www.hpa.gov.tw/Pages/Detail.aspx?nodeid=1061&pid=6071). Most important of all, the above-mentioned prediction model was validated in another cohort with an acceptable discriminatory ability.

Several hematological inflammatory markers, such as the C-reactive protein-to-albumin ratio, modified Glasgow Prognostic Score, neutrophil to lymphocyte ratio, and platelet-to-lymphocyte ratio, have been used to predict IC toxicity in HNC [11]. The plausible mechanism for the toxicity is attributed to poor nutrition, inflammation, and immune suppression. However, a significant drawback was that body mass index (BMI) was the sole body composition factor in their statistical analyses. Besides BMI, body composition factor, like a low SMI or LBM, has been reported to yield adverse effects on chemotherapy dose-limiting toxicity. More specifically, a low SMI is suggested to be related to a chemotherapy dose reduction of ≥ 50% due to neutropenia or nephrotoxicity, a treatment postponement ≥ 4 days in the case of bone marrow suppression, or definitive chemotherapy termination after the first or second chemotherapy cycles in HNC patients receiving definitive chemoradiotherapy [12]. Our previous study has documented the adverse prognostic effects of sarcopenia on overall survival (aHR: 1.74; 95% CI: 1.14–2.67) and disease-specific survival (aHR: 1.67; 95% CI: 1.04–2.67) in oral cancer patients, and the negative effect was even more significant among patients aged < 60 years [18].

Our study revealed that the chemotherapy dose per kilogram LBM, like docetaxel per kilogram LBM more than 2.52 mg/kg could be a significant toxicity predictor in male HNC patients. The primary toxicity mechanism might be chemotherapy overdose in specific populations, such as in sarcopenia or myopenia [21]. Most chemotherapy dosages are determined by BSA, while some have a fixed or capped dose (e.g., carboplatin). Given the BSA-based dose calculation formulae, patients having a similar BSA but with a lower LBM were treated with a higher dose of chemotherapy per LBM. This phenomenon was more prominent in docetaxel than cisplatin cases, which was expected from a pharmacokinetic perspective. Increased adipose tissue denotes a higher distribution for and uptake of lipid-soluble drugs, such as docetaxel, and a lower distribution for water-soluble drugs, such as cisplatin [22–24]. This difference explains why patients with a smaller muscle proportion and a higher adipose tissue proportion were more prone to docetaxel toxicities, as both the protective effect of muscle and the negative effect of excess adipose tissue are contributory [25]. Due to limited number of female patients and events, the prediction model was not developed.

Toxicities are sometimes indicative of adequate chemotherapy dose, which may be associated with better outcomes in solid tumor cancers, such as gastric cancer, non-small cell lung cancer, and prostate cancer [26–28]. However, toxicities could also result in postponement or termination of chemotherapy, resulting in worse outcomes [12]. In our study, HNC patients with significant toxicities during induction chemotherapy were not associated with better response rates (58.8% in patients without toxicity event vs. 50% in patients with toxicity event, *P* = 0.389; Supplementary table 7). The benefit of the toxicity event, a proxy of adequate chemotherapy dose per cycle, might be offset by insufficient relative dose intensity (RDI) [9]. Using our risk score, prophylactic protocol, like granulocyte-colony stimulating factor injection or intensive care, could be applied to the high-risk group in order to prevent DLT events and achieve acceptable RDI.

Our study has several limitations. First, we performed a retrospective study with the abovementioned data extracted from our electronic medical records. Although most laboratory data have been rechecked within one week of IC, some variations still existed. Second, this retrospective study was designed to find new predictors instead of creating a new formula for chemotherapy dose modifications in clinical scenarios. Chemotherapy dose modifications require further large-scale, high-quality observational studies and clinical trials, such as cabazitaxel dose modification in prostate cancer [29]. Third, the SMI or CSA at C3 was derived from head and neck CT, MRI, or PET scan images, rendering the possibility of inaccurate estimates. Nevertheless, previous literature has validated the interchangeability of CT- or MRI-derived imaging biomarkers of SMI [19]. Fourth, the DLT event rate was higher in the validation cohort. The difference was due to missing data about DLT events before 2021. We launched a quality improvement for adverse effect monitoring in HNC with chemotherapy since 2021. Thereafter, the records of DLT events were accurate. Fifth, a large variation of body composition factors and chemotherapy per kilogram LBM between the male and female patients were noted. Stratified analysis was performed, and risk score for male HNC patients alone was established due to unequal sample size between male and female patients. Finally, the prediction model for male HNC patients was validated in another cohort with an acceptable discriminatory ability. It deserved another HNC cohort in western countries for external validation and generalization.

## Conclusions

High docetaxel per kilogram LBM (> 2.52 mg/kg), pre-treatment GPT > 40 U/L, and history of chronic liver disease were significant risk factors for DLT events in male HNC patients treated with taxane and platinum-based induction chemotherapy. Before an adjusted chemotherapy dose through LBM or other body composition factors becomes available, identifying high-risk patients could help physicians in clinical care, prevent severe/fatal complications and improve the relative dose intensity of chemotherapy among male HNC patients treated with induction chemotherapy.

## Supplementary Information

**Additional file 1: Supplementary table 1.** Intra-class correlation for different images at initial recruitment. **Supplementary table 2.** Variation of body composition factors and chemotherapy dose. **Supplementary table 3.** Cutoff points of chemotherapy per kilogram lean body mass for male and female head and neck patients. **Supplementary table 4.** Beta coefficient of independent predictors for DLT events in male head and neck cancer patients. **Supplementary table 5.** Demographic data of the validation cohort (*n* = 34). **Supplementary table 6.** Frequency of toxicity events after the first cycle of induction chemotherapy in the validation cohort (*n* = 34). **Supplementary table 7.** Toxicity event during the 1^st^ cycle and response rate (*n*=125).

**Additional file 2: Supplementary Figure 1.** Example of skeletal muscle measurements at the third cervical vertebral (C3) level on axial neck CT (A) and MRI (B) images. The paravertebral muscles are shown in green. The left sternocleidomastoid muscle is delineated in red, and the right sternocleidomastoid muscle in yellow.

## Data Availability

The datasets generated and analyzed of the current study are available from the corresponding author on reasonable request after the approval of our IRB.

## Abbreviations

IC: Induction chemotherapy
FN: Febrile neutropenia
AKI: Acute kidney injury
LBM: Lean body mass
DLT: Dose-limiting toxicity
HNC: Head and neck cancer
SMI: Skeletal muscle index
aOR: Adjusted odds ratio
CI: Confidence interval
GOT: Glutamic oxaloacetic transaminase
GPT: Glutamic pyruvic transaminase
CSA: Cross-sectional area
CT: Computerized tomography
MRI: Magnetic resonance imaging
PET: Positron emission tomography
ANC: Absolute neutrophil count
ULN: Upper limit of normal
RECIST: Response Evaluation Criteria in Solid Tumors
