# Supplementary material for: Chemotherapy dose per kilogram lean body mass increased dose-limiting toxicity event in male head and neck cancer with taxane and platinum-based induction therapy
Source: BMC Cancer. 2022 Oct 21;22:1084. doi: 10.1186/s12885-022-10152-y (PMC9587609; doi:10.1186/s12885-022-10152-y)
Supplement: Supplementary file 1 — Additional file 1: Supplementary table 1. Intra-class correlation for different images at initial recruitment. Supplementary table 2. Variation of body composition factors and chemotherapy dose. Supplementary table 3. Cutoff points of chemotherapy per kilogram lean body mass for male and female head and neck patients. Supplementary table 4. Beta coefficient of independent predictors for DLT events in male head and neck cancer patients. Supplementary table 5. Demographic data of the validation cohort (n = 34). Supplementary table 6. Frequency of toxicity events after the first cycle of induction chemotherapy in the validation cohort (n = 34). Supplementary table 7. Toxicity event during the 1st cycle and response rate (n=125). [file 12885_2022_10152_MOESM1_ESM.docx]

| Supplementary table 1. Intra-class correlation for different images at initial recruitment | | |
| --- | --- | --- |
| Variables | Intra-class correlation | 95% confidence interval |
| CT | 0.891 | 0.836-0.926 |
| MRI | 0.915 | 0.878-0.94 |
| PET | 0.94 | 0.894-0.966 |
| Overall | 0.94 | 0.923-0.953 |

| Supplementary table 2. Variation of body composition factors and chemotherapy dose | | | | |
| --- | --- | --- | --- | --- |
| Variables | Maximal value | Minimal value | Maximal value/  Minimal value |  |
| BMI | 36.7 | 14.6 | 2.51 |  |
| LBM | 57.7 | 23.8 | 2.42 |  |
| SMI | 68.8 | 26.5 | 2.60 |  |
| Docetaxel per kilogram LBM | 4.14 | 1.53 | 2.71 |  |
| Cisplatin per kilogram LBM | 4.14 | 1.53 | 2.71 |  |

Abbreviations: BMI, body mass index; LBM, lean body mass; SMI, skeletal muscle index

| Supplementary table 3. Cutoff points of chemotherapy per kilogram lean body mass for male and female head and neck patients | | |
| --- | --- | --- |
| Variables | Males | Females |
| *Docetaxel per kilogram lean body mass* | | |
| Lower third | <2.38 | <3.22 |
| Middle third | >=2.38 and<=2.52 | >=3.22and <=3.47 |
| Upper third | >2.52 | >3.47 |
| *Cisplatin per kilogram lean body mass* | | |
| Lower third | <2.37 | <3.22 |
| Middle third | >=2.37 and <=2.53 | >=3.22 and <=3.47 |
| Upper third | >2.53 | >3.47 |

| Supplementary table 4. Beta coefficient of independent predictors for DLT events in male head and neck cancer patients | | | |
| --- | --- | --- | --- |
| Variables | Beta coefficient | Adjusted odds ratio | P value |
| Docetaxel per kilogram lean body mass | | | |
| Lower third |  | 1 |  |
| Middle third | 0.421 | 1.52 (0.57-4.01) | 0.4 |
| Upper third | 1.158 | 3.18 (1.25-8.09) | 0.015 |
| Pre-treatment GPT>40 | 0.959 | 2.61 (1.03-6.64) | 0.044 |
| History of chronic liver disease | 1.382 | 3.98 (1.03-15.46) | 0.046 |

Abbreviation: DLT events, dose-limiting toxicity events.

| Supplementary table 5. Demographic data of the validation cohort (n = 34) | |
| --- | --- |
| Age (mean ± SD) | 54.6±7.3 |
| Gender |  |
| Male | 32 (94.1%) |
| Female | 2 (5.9) |
| Lean body mass (mean ± SD) | 39.7±5.1 |
| Skeletal muscle index(mean ± SD) | 46.3±6.0 |
| Docetaxel per kilogram lean body mass (mean ± SD) | 2.67±0.21 |
| Cisplatin per kilogram lean body mass (mean ± SD) | 2.67±0.2 |
| Clinical T classification |  |
| cT1-2 | 12(35.3%) |
| cT3-4 | 22 (64.7%) |
| Clinical N classification |  |
| cN0-1 | 14 (41.2%) |
| cN2-3 | 20 (58.8%) |
| Tumor subsites |  |
| Oral cavity | 4 (11.8%) |
| Oropharynx | 18 (52.9%) |
| Hypopharynx/Larynx | 10 (29.4%) |
| Others | 2 (5.9%) |
| Comorbidities |  |
| Chronic liver disease | 10 (29.4%) |
| Hypertension | 4 (11.8%) |
| Diabetes | 4 (11.8%) |
| Induction chemotherapy regimen |  |
| Docetaxel + Cisplatin | 34 (100%) |

| Supplementary table 6. Frequency of toxicity events after the first cycle of induction chemotherapy in the validation cohort (n = 34) | |
| --- | --- |
| Grade III + hematological adverse event | 11 (32.4) |
| Neutropenia | 11 (32.4) |
| Anemia | 1(2.9) |
| Thrombocytopenia | 1 (2.9) |
| Grade III + non-hematological adverse event | 2 (5.9) |
| Acute liver injury | 0) |
| Acute kidney injury | 2 (5.9) |
| Grade III + hematological and non-hematological adverse event | 11 (32.4) |
| Subsequent chemotherapy dose reduction more than 50% | 0 |
| Postponement of chemotherapy > = 4 days | 2 (5.9) |
| Death during or after chemotherapy | 2 (5.9) |
| Overall DLT events | 13 (38.2) |
| Abbreviation: DLT events, dose-limiting toxicity events. |  |

| Supplementary table 7. Toxicity event during the 1^st^ cycle and response rate (n=125)* | | | | |
| --- | --- | --- | --- | --- |
| Variables | Complete response | Partial response | Stable disease or progression disease | Pvalue |
| With toxicity event (n=28) | 5(17.9%) | 9(32.1%) | 14(50%) | 0.389 |
| Without toxicity event (n=97) | 12(12.4%) | 45(46.4%) | 40(41.2%) |  |

*125 HNC patients were available for response evaluation.
