# Supplementary material for: Chemotherapy dose per kilogram lean body mass increased dose-limiting toxicity event in male head and neck cancer with taxane and platinum-based induction therapy
Source: BMC Cancer. 2022 Oct 21;22:1084. doi: 10.1186/s12885-022-10152-y (PMC9587609; doi:10.1186/s12885-022-10152-y)
Supplement: Supplementary file 2 — Additional file 2: Supplementary Figure 1. Example of skeletal muscle measurements at the third cervical vertebral (C3) level on axial neck CT (A) and MRI (B) images. The paravertebral muscles are shown in green. The left sternocleidomastoid muscle is delineated in red, and the right sternocleidomastoid muscle in yellow. [file 12885_2022_10152_MOESM2_ESM.docx]

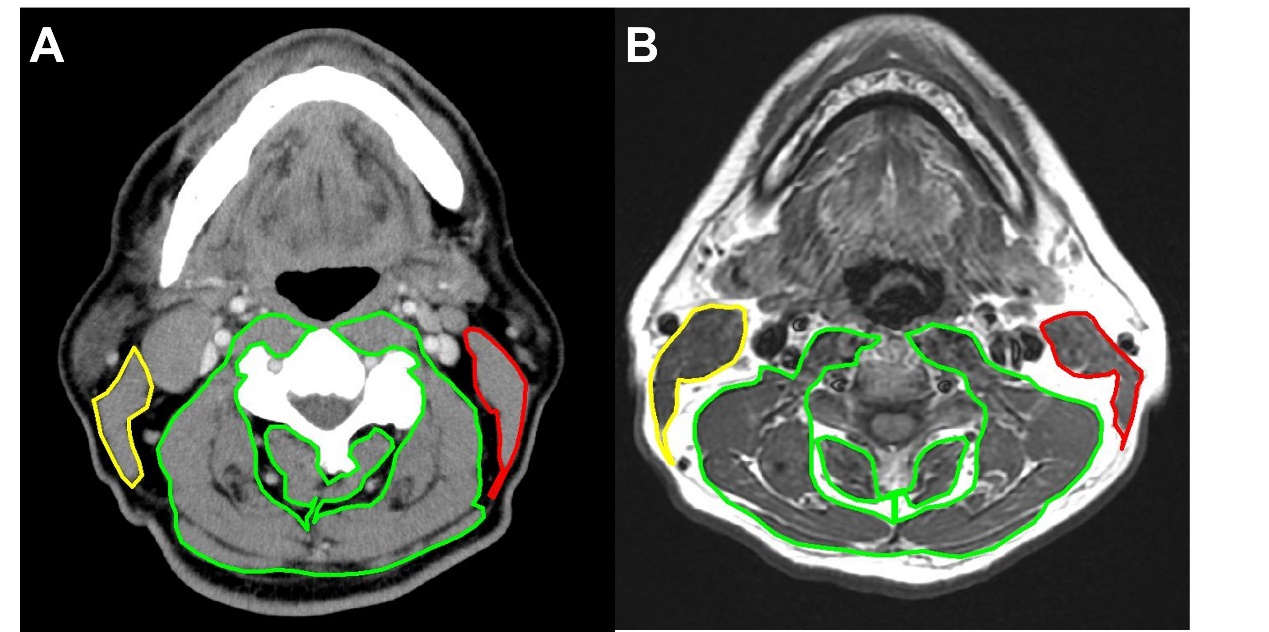


Supplementary Figure 1. Example of skeletal muscle measurements at the third cervical vertebral (C3) level on axial neck CT (A) and MRI (B) images. The paravertebral muscles are shown in green. The left sternocleidomastoid muscle is delineated in red, and the right sternocleidomastoid muscle in yellow.
